# Fabrication of Naturally Derived Double-Network Hydrogels With a Sustained Aspirin Release System for Facilitating Bone Regeneration

**DOI:** 10.3389/fchem.2022.874985

**Published:** 2022-03-28

**Authors:** Wenfeng Zhu, Rui Chen, Weiheng Wang, Yi Liu, Changgui Shi, Songjun Tang, Guoke Tang

**Affiliations:** ^1^ Department of Orthopedics, Shanghai Post and Telecommunication Hospital, Shanghai, China; ^2^ Department of Orthopedics, Second Affiliated Hospital of Naval Medical University, Shanghai, China; ^3^ Department of Orthopedics, Shanghai General Hospital, Shanghai Jiaotong University, Shanghai, China

**Keywords:** DN hydrogel, bone regeneration, drug delivery, aspirin, GelMA

## Abstract

Continuous efforts on pursuit of effective drug delivery systems for engineering hydrogel scaffolds is considered a promising strategy for the bone-related diseases. Here, we developed a kind of acetylsalicylic acid (aspirin, ASA)–based double-network (DN) hydrogel containing the positively charged natural chitosan (CS) and methacrylated gelatin (GelMA) polymers. Combination of physical chain-entanglement, electrostatic interactions, and a chemically cross-linked methacrylated gelatin (GelMA) network led to the formation of a DN hydrogel, which had a suitable porous structure and favorable mechanical properties. After *in situ* encapsulation of aspirin agents, the resulting hydrogels were investigated as culturing matrices for adipose tissue–derived stromal cells (ADSCs) to evaluate their excellent biocompatibility and biological capacities on modulation of cell proliferation and differentiation. We further found that the long-term sustained ASA in the DN hydrogels could contribute to the anti-inflammation and osteoinductive properties, demonstrating a new strategy for bone tissue regeneration.

## Introduction

Bone defects from the congenital bone diseases, limb trauma, tumors, and infectious diseases can cause many severe problems and reduce the life quality of humans (Service, 2000; Kun Zhang et al., 2019; Wang et al., 2019; Cheng et al., 2021). The conventional surgical operations for bone therapy are generally limited because of the insufficient transplantation materials (Dimitriou et al., 2011). As a gold standard method, autologous transplantation has still been hindered due to the lack of autologous bone sources and potential risks of postoperative infection and nerve injury (Langer and Vacanti, 1993; da Silva et al., 2007; Schmitt et al., 2012; Miller and Chiodo, 2016; Chiodo et al., 2010). To address these troubles, a designable strategy of bionic hydrogel scaffolds based on advance exogenous progenitor cells and controllable release of bioactive drugs or factors within the networks is significantly important for effective therapy of bone defect (Stratton et al., 2016; Armiento et al., 2020; Wang et al., 2020; Shang et al., 2021; Tang et al., 2021). The hydrogel scaffolds mainly comprise synthetic hydrogels or naturally derived hydrogels, which have been widely utilized to exploit the bone regenerative capacities on biocompatibility, biodegradability, network architecture, and mechanical properties to emulate extracellular matrices for cell viability, adhesion, growth, proliferation, and differentiation in bone regeneration fields (Malafaya et al., 2007; Seliktar, 2012; Sun et al., 2012; Dou et al., 2020; Yu et al., 2020). Synthetic hydrogels including the typical polyethylene glycol (PEG), poly(vinyl alcohol) (PVA), poly(ε-caprolactone) (PCL), poly(acrylic acid) (PAA), poly(lactic acid) (PLA), and polycarbonate urethane (PU) possess designable structures and tunable properties (e.g., degradation time, mechanics, and machinability) for fabrication of various multifunctional scaffold products. However, the suspected residues such as contaminants, unreacted reagents, surplus monomers, catalysts, and other byproducts are difficult to completely remove during the preparation process, thus jeopardizing biosafety (Athanasiou et al., 1996; Zhang et al., 2012; Sennakesavan et al., 2020; Zhou et al., 2020; Zhu et al., 2022). In comparison, naturally derived hydrogels inherently possess excellent biocompatibility and biodegradability for cell growth to construct biological scaffolds. Chitosan, gelatin, alginate, collagen, hyaluronan, and agarose are naturally derived biopolymers, with easy availability of renewable resources from the animals, plants, marine organisms, and microorganisms (Kashirina et al., 2019; Ranganathan et al., 2019; Zhai et al., 2019; Tang et al., 2020; Zou et al., 2021), wherein the biocompatible gelatin with a peptide sequence (Arg–Gly–Asp, RGD) and similar collagen composition has been widely used in the fabrication of tissue engineering scaffolds for cell adhesion, proliferation, and differentiation (Moreira et al., 2019). However, the physical gelatin hydrogel itself or extensively used chemical cross-linked methacrylated gelatin (GelMA) hydrogels are often weak and brittle in mechanical properties, precluding their bioapplications as load-bearing scaffolds. Another representative chitosan (CS) is a cationic polysaccharide widely used in the construction of drug carriers and tissue engineering scaffolds. This sole alkaline CS polymer is enzymatically degradable for satisfying the implanted scaffolds, and its physical chain-entanglement network, rigid backbone, and a number of natural amino groups could provide the obvious electrostatic interactions with carboxyl-modified drugs or bioactive factors; in this case, the CS-based hydrogels can be used as an intelligent carrier to tailor the drug loading and delivery behaviors (Yang et al., 2018; Abadi et al., 2021; Feng and Wang, 2022; Jiang et al., 2022; Peers et al., 2022; Yang et al., 2022; Zhang et al., 2022). However, CS-based physical hydrogels are also poor in mechanical property. Therefore, development of the naturally derived composite hydrogels is an effective method for meeting cell support and mechanical stability in the regenerative medicine. Although many progresses had been made in the past few decades, appropriate incorporation of therapeutics into hydrogels to efficiently promote bone regeneration is still challenging.

Acetylsalicylic acid (ASA) is a widely used nonsteroidal anti-inflammatory drug that can affect multiple biological processes and the local microenvironment of mesenchymal stem cells in MSC-mediated bone regeneration. It can elevate the osteogenic differentiation through activation of osteoblasts and inhibition of osteoclasts (Yamaza et al., 2008), but its rapid dissolution nature and short half-life greatly limit its clinical applications (Bliden et al., 2016), which urgently require a suitable scaffold and drug delivery system to achieve sustainable transportation at the site of bone repair. A recent study reported an aspirin-based tetra-PEG hydrogel with a sustained release system to promote the osteogenesis performance, but simultaneously satisfying mechanics and cell proliferation is necessarily improved in bone regeneration (Yunfan Zhang et al., 2019).

Here in this study, we designed and prepared an ASA-encapsulated GelMA-CS DN hydrogel with therapeutic effect on bone regeneration. Both naturally derived gelatin and CS polymers exhibited strong biocompatibility associated with the extracellular matrix for facilitating cell viability, adhesion, growth, and proliferation. This DN hydrogel possessed suitable network pores and moderate mechanical properties for the construction of smart drug scaffolds. By means of the electrostatic interactions between the oppositely charged groups, the drug agents of carboxyl-modified ASA were *in situ* encapsulated and well-distributed within the amine-abundant DN hydrogels, which endowed the biocompatible and biodegradable hydrogel with sustained aspirin delivery locally and elucidated the dose-dependent therapeutic efficiency by promoting anti-inflammation, osteogenic differentiation, and bone regeneration. The resulting hydrogels have been broadly utilized as scaffolds for therapeutic agents in tissue engineering.

## Materials and Methods

### Materials

Aspirin (99%, J&K), gelatin (80–100 kDa, J&K), and CS (ca. 10 kDa, degree of deacetylation >90%, viscosity: 45 mPa s) was purchased from Shandong Jinhu Company. All other reagents were purchased from Sigma-Aldrich and used as received without further purification. Adipose tissue–derived stromal cells (ADSCs) were supplied by China Infrastructure of Cell Line Resource.

### Preparation of GelMA-CS and ASA-Loaded GelMA-CS DN Hydrogels

The GelMA-CS DN hydrogel was simply prepared by adding the stock solution of CS (3 wt%, 1 ml) to the stock solution of GelMA (15 wt%, 1 ml) containing the photoinitiator followed by UV irradiation. The ASA-loaded GelMA-CS DN hydrogel was obtained by simultaneously mixing the appropriate amount of ASA (10 or 100 µg/ml) into the aforementioned hydrogel solutions under UV irradiation at room temperature.

### Scanning Electron Microscopy (SEM) Observation

SEM images of DN hydrogels were obtained at an acceleration voltage of 5 kV on a JSM-6700F microscope (JEOL, Japan). The freeze-dried samples were sputter-coated with a thin layer of Pt for 90 s to prepare the conductive sample before testing.

### Rheology of DN Hydrogels

Rheological characteristics of GelMA-CS and GelMA-CS@ASA DN hydrogels were conducted on a rheometer (Thermo Haake Rheometer, Newington, NH, United States). During the experiments, the hydrogels were spread on a parallel plate (25 mm) and sealed with silicone oil to prevent solvent evaporation. Frequency scan: 0.1–100 rad/s. Temperature: 25°C.

### Compressive Property

The compressive profiles of GelMA-CS and GelMA-CS@ASA DN hydrogels were measured using an Instron 3365 testing machine (Instron Co., Norwood, MA, United States). The hydrogel samples were cut into cylinders for compressive testing with a beam velocity of 1 mm/min.

### *In Vitro* ASA Release From the Hydrogels

After preparation of the GelMA-CS@ASA hydrogels with a diameter of 20 mm and height of 4 mm in a container, the ASA-contained hydrogel was immersed into PBS solutions at 37°C, which were collected at appointed intervals of time. The collected solutions at various times were tested using UV–visible spectroscopy.

### Cytotoxicity Assay

Cytotoxicity was carried out using the Cell Counting Kit-8 assay. The ADSCs (2 × 10^3^/100 µl/well) were first seeded in 96-well microplates and incubated at 37°C in 5% CO_2_ for 12 h to obtain a monolayer of cells. Then, hydrogel samples were added to each well and incubated for the predetermined time. After 1, 2, 3, 4 ,and 5 days of incubation, the cell culture medium was removed and then 100 µl of fresh culture medium and 10 µl of CCK-8 were added to the 96 wells and incubated for 2 h. Finally, the absorbance was read at 450 nm on a microplate reader (Thermo Fisher Scientific, Waltham, MA, United States). Cell viability (%) was calculated using the following equation:

$$\mathrm{Cell}\;\mathrm{viability}\left(\%\right)=\left[\left(A_{\mathrm{sample}}-A_{\mathrm{blank}}\right)/\left(A_{\mathrm{control}}-A_{\mathrm{blank}}\right)\right]\times 100\%.$$

The data represented the mean of five independent experiments and were expressed as mean ± SD.

### Live/Dead Staining Assay

Cell live and dead viability was determined by using a live/dead viability assay, according to the manufacturer’s instruction. The staining reagent mixture, a red fluorescent propidium iodide (PI) stain and a green fluorescent (AM) stain, was added to the reaction mixture and incubated in the dark at a room temperature for 15 min. The corresponding fluorescence emission of ADSCs was then assessed using confocal laser scanning microscopy (CLSM).

### Alizarin Red S (ARS) Staining

After 14 days of osteogenic induction, ADSCs were fixed and rinsed in the same way as ALP staining by 1% of the ARS (Sigma-Aldrich) dye for 15 min at room temperature, which were then rinsed by PBS solutions three times. Finally, the stained ADSCs were dried and photographed. ImageJ2 (NIH, United States) was utilized to measure the stained areas for semi-quantitative analysis (*n* = 3).

### Semi-Quantitative RT-PCR

The total RNA was extracted using TRIzol reagent (Invitrogen), and cDNA was prepared from 200 ng of total RNA by using the RevertAid™ H Minus First Strand cDNA Synthesis Kit (Thermo Fisher Scientific, Waltham, MA). Template PCRs were performed after 33 cycles of amplification with the adjusted annealing temperature. The primers sequences are listed in Table 1.

**TABLE 1:** Sequences of quantitative polymerase chain reaction primers.

Gene	Forward Primer (5′‐3′)	Reverse Primer (3′‐5′)
*Runx2*	ATG​CTT​CAT​TCG​CCT​CAC​AAA​C	CCA​AAA​GAA​GTT​TTG​CTG​ACA​TGG
*ALP*	CCCAAAGGCTTCTTCTTG	CTGGTAGTTGTTGTGAGC
*GAPDH*	ACC​ACA​GTC​CAT​GCC​ATC​AC	TCC​ACC​ACC​CTG​TTG​CTG​TA

RUNX2, runt-related transcription factor 2; ALP, alkaline phosphatase; GAPDH, glyceraldehyde-3-phosphate dehydrogenase.

### Statistical Analysis

All results were presented as mean and standard deviation (mean ± S.D.) of 3–6 independent experiments. The statistics were analyzed using SPSS software (ver. 13.0; SPSS Inc., United States). *P* < 0.05 was considered to be statistically significant.

## Results and Discussion

### Preparation and Characterization of GelMA-CS and GelMA-CS@ASA DN Hydrogels

**FIGURE 1:**
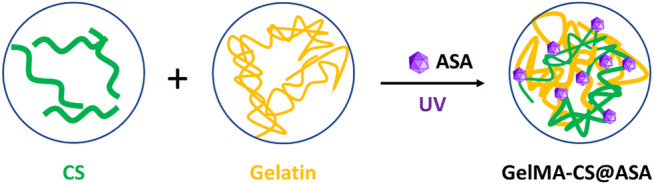
Schematic illustration of the fabricated ASA-encapsulated GelMA-CS DN hydrogels.

The schematic illustration of GelMA-CS@ASA DN hydrogels is demonstrated in Figure 1. Under UV irradiation, the cross-linking gelatin network was first rapidly formed *in situ* to facilitate gel formation. In view of the intrinsic properties of physically electrostatic interactions and chain-entanglement effects between the gelatin and CS chains, a second noncovalent robust network was gradually formed to enhance the mechanical stiffness. Meanwhile, the therapeutic ASA agents could be well-encapsulated and distributed around the gelatin and CS chains by electrostatic interactions within the hydrogels, which presented a sustained release behavior to prolong the maintenance time of effective drug concentration *in situ* for bone regeneration. As a sole alkaline polysaccharide, CS possessed a number of natural amino groups that could generate electrostatic interactions with carboxyl-modified ASA drugs to guide the controlled drug release. In addition, gelatin had a peptide sequence (Arg–Gly–Asp, RGD) with excellent gelling performance and favorable advantages in terms of cell attachment, proliferation, and differentiation for application in tissue engineering.

**FIGURE 2:**
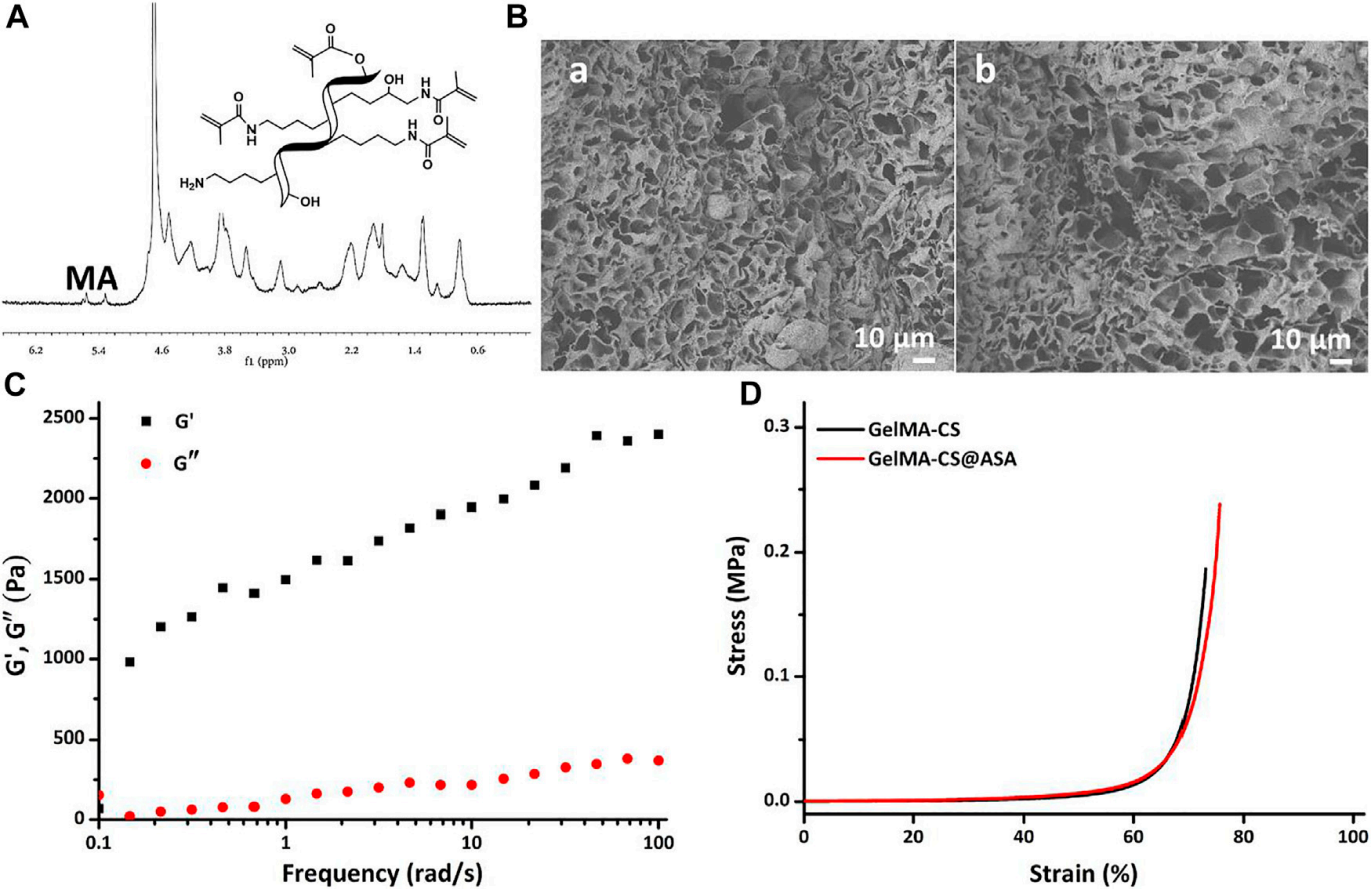
**(A)** ^1^H NMR spectrum of GelMA. **(B)** SEM images of **(a)** GelMA-CS and **(b)** GelMA-CS@ASA hydrogels. **(C)** Rheological profile of the GelMA-CS@ASA hydrogel. **(D)** Compressive curves of GelMA-CS hydrogels with or without ASA loaded.

Figure 2A showed the ^1^H NMR spectrum of GelMA with a simple and effective anhydride reaction of gelatin in solutions. The morphologies of DN hydrogels were observed by SEM images in Figure 2B, which showed the similar inner porous networks of GelMA–CS and GelMA–CS@ASA DN hydrogels. No significant difference was found between these two groups (Figure 2B). Hydrogel scaffolds with suitable pore size and porosity could allow host cell infiltration and the exchange of nutrition and metabolic waste, which provided more chance to enable the sustained release of the encapsulated ASA drugs, cell entry, and substance exchange intra–extra of the hydrogels. Similar to the previous literatures, we encapsulated ASA in the GelMA-CS hydrogel without changing its morphological architecture, indicating that the introduction of electrostatic interaction between the carrier and drug molecules can significantly improve drug release kinetics (Mauri et al., 2017). We then carried out rheological experiments to confirm the formation of the GelMA-CS@ASA hydrogel and investigate its mechanical property. As shown in Figure 2C, the storage modulus (G′) obviously surpassed the loss modulus (G″) throughout the whole frequency range, confirming the formation of a hydrogel even with the addition of ASA drug molecules. In addition, these hydrogels also possessed the similar compressive stress in Figure 2D that was beyond the traditional gelatin hydrogels, revealing the importance of the rigid CS backbone on improving the mechanical strength of the DN hydrogel. These results indicated that ASA loading did not alter and affect the microarchitectures and mechanical performances of DN hydrogel scaffolds.

**FIGURE 3:**
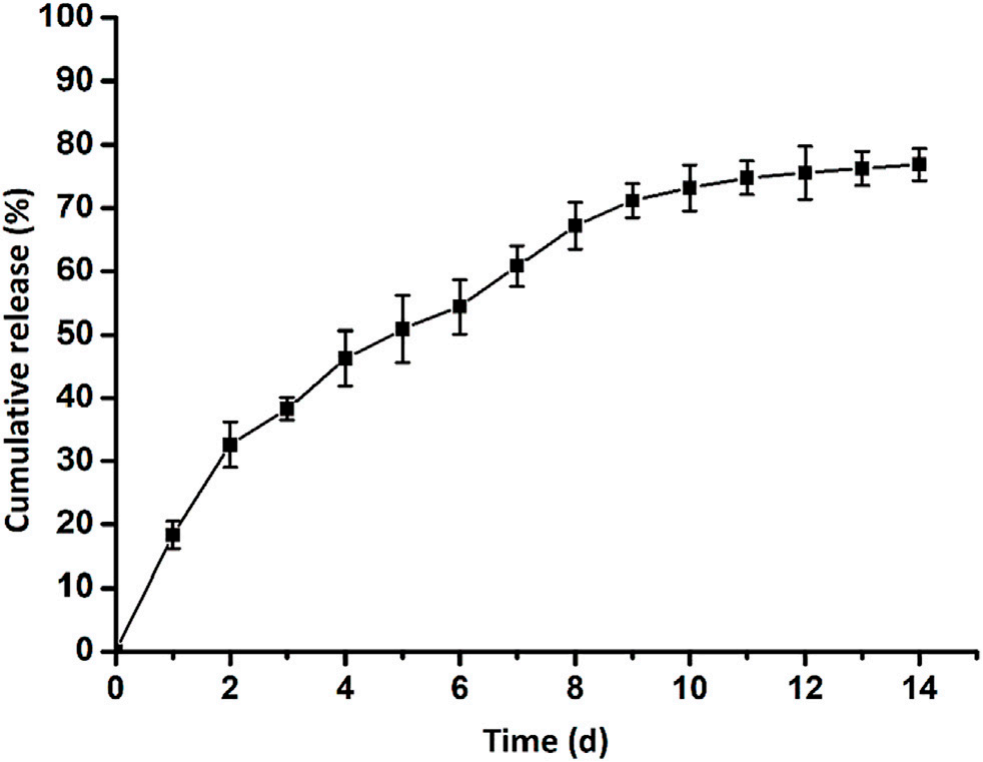
Release behavior of aspirin from the GelMA-CS@ASA DN hydrogel.

Furthermore, we detected the release profile of ASA loaded in the GelMA-CS@ASA DN hydrogel *in vitro*. As shown in Figure 3, a constant and sustained release of the ASA drug was observed up to 14 days, which was due to the electrostatic interactions of CS with carboxyl-modified ASA drugs to control drug release. In the first 2 days, a cumulative release of the ASA drug quickly reached round 33%. This initial burst ASA release could afford sufficient stimuli to meet the requirement of the defect areas. Then, the release rates of ASA approached its plateaus on the 6th day, and the cumulative release rate of ASA reached nearly 80% on the 14th day, which indicated a sustained ASA release profile from the DN hydrogel.

### Cell Viability and Proliferation

**FIGURE 4:**
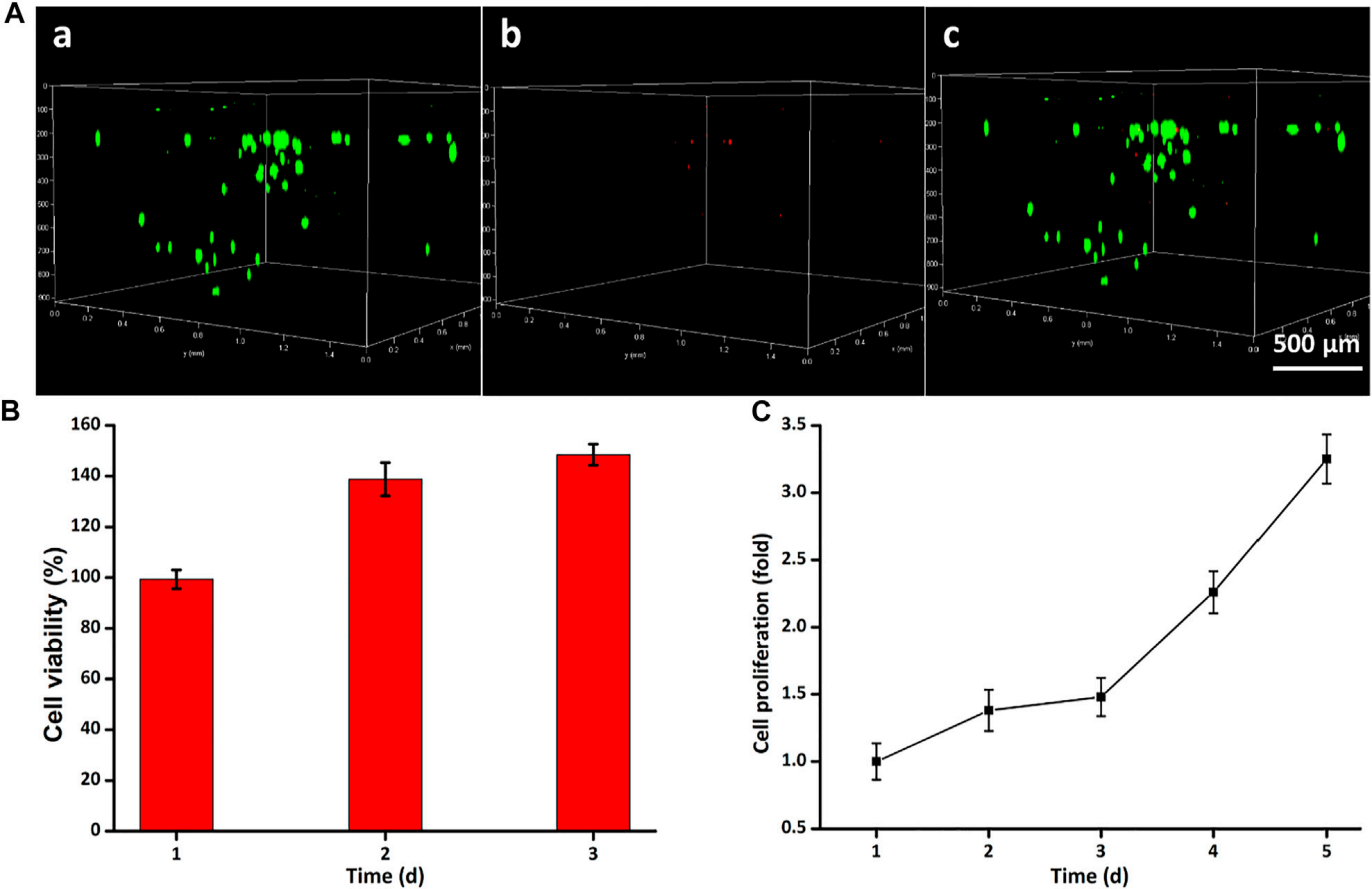
Cytotoxicity of GelMA-CS@ASA hydrogels *in vitro*. **(A)** Live/dead staining of ADSCs. Cells in green manifest living ADSCs, while cells in red manifest dead ones. **(B)** Cell viability and **(C)** proliferation was detected by using the Cell Counting Kit-8 after cultivation for various time periods.

Cell live–dead staining is a green fluorescent labeling technique using Calcein-AM as a dye for the identification of cell death/live status, which was used to intuitively assess the biocompatibility. After coculturing with the hydrogel scaffolds for 24 h, most cells could crawl along the 3D printed implant pillars to form a three-dimensional solid adhesive morphology. Figure 4A confirmed that hydrogels exerted low cytotoxicity on ADSCs and ADSCs maintained a good survival status. Particularly, there were even fewer dead cells stained with PI than in the TS group in some areas, manifesting the high viability in the early stages of 24 h. Quantitatively, the GelMA-CS@ASA DN hydrogel could also promote cell viability and cell growth. To investigate if this hydrogel was able to support cell growth and proliferation, a long-term proliferation of the CCK-8 assay was conducted after culture with the GelMA-CS@ASA hydrogel for 5 days. Figure 4B further testified the excellent biocompatibility of hydrogel scaffolds at these time points with no significant difference among the groups. The cell proliferation rate was slightly increased in the initial 3 days after coculturing with the DN hydrogel and showed a significant increase on the 5th day of incubation, as observed in Figure 4C, revealing favorable cell viability, growth, and proliferation capacity of this kind of DN hydrogel.

### Osteogenic Differentiation of ADSCs in the DN Hydrogel Scaffolds *In Vitro*

**FIGURE 5:**
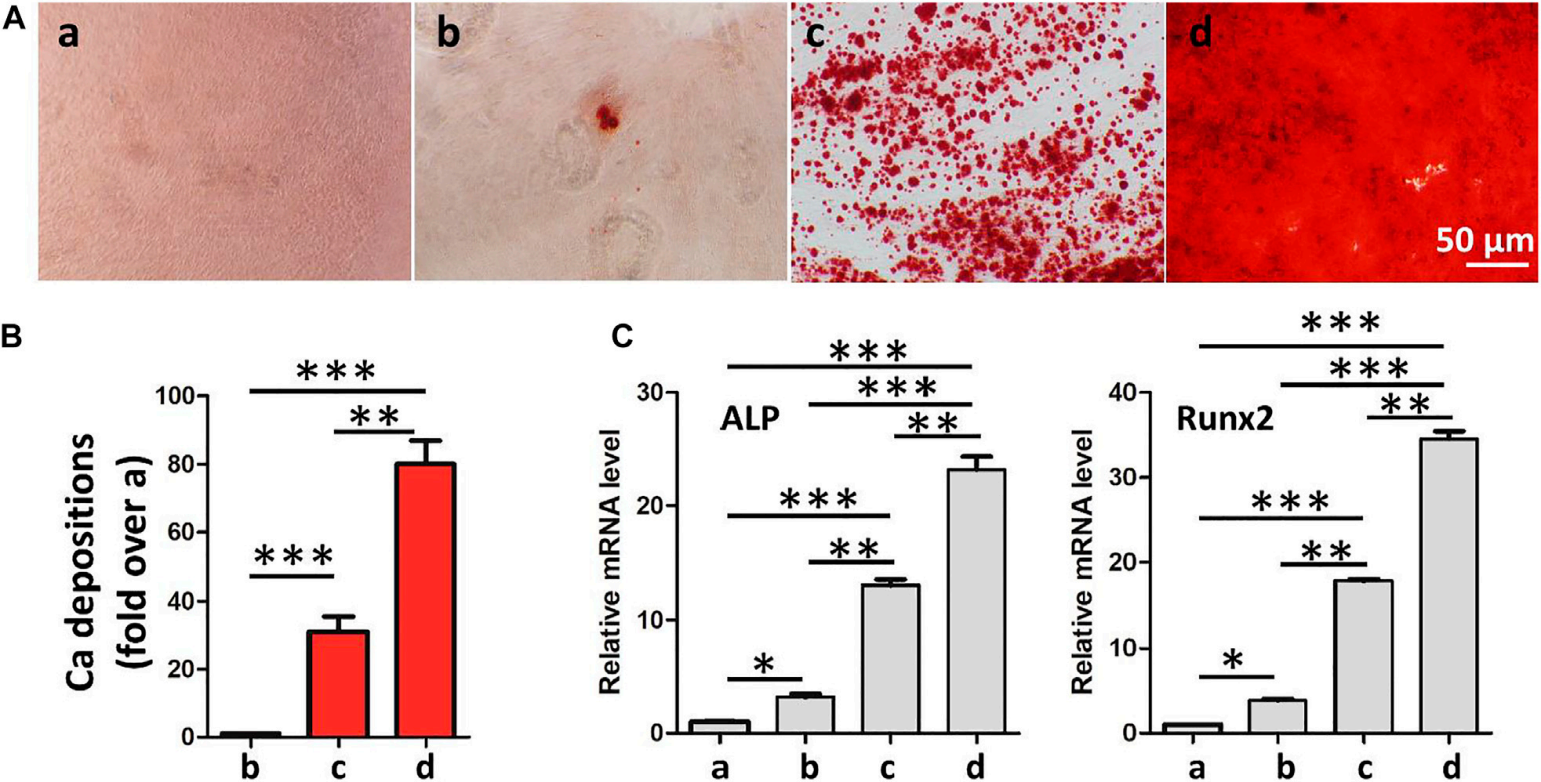
Effects of GelMA-CS@ASA DN hydrogels on the ADSC osteogenesis. **(A)** Osteogenic differentiation detection based on Alizarin Red. **(B)** Quantification of calcification depositions. **(C)** mRNA analysis of osteogenic markers of ALP and Runx2. Statistically significant differences in comparison with **(a)** control untreated cells, **(b)** GelMA-CS hydrogel, **(c)** GelMA-CS@ASA (ASA, 10 μg/ml) hydrogel, and **(d)** GelMA-CS@ASA (ASA, 100 μg/ml) hydrogel. ***p < 0.001, **p < 0.01, *p < 0.05.

Ideal engineering bone repair scaffolds should enhance the osteogenic differentiation of ADSCs. To reveal the effect of hydrogel scaffolds on the osteogenic differentiation *in vitro*, we seeded the ADSC cells on the control, GelMA-CS, GelMA-CS@ASA (10 μg/ml), and GelMA-CS@ASA (100 μg/ml) DN hydrogels. ARS staining and qPCR assay were used to examine their osteoinduction abilities. As shown in Figure 5A, the GelMA-CS@ASA DN hydrogel could significantly increase the calcification nodule formation capacity of ADSCs after 14 days of osteogenic induction. Real-time PCR showed that the expression of osteogenesis-related genes in the GelMA-CS, GelMA-CS@ASA (10 μg/ml), and GelMA-CS@ASA (100 μg/ml) DN hydrogels was higher than that in the control group (Figures 5B,C). The mRNA levels of osteogenic markers of typical ALP and Runx2 on the cell inoculated in the GelMA-CS@ASA DN hydrogels were higher than those in the GelMA-CS groups, suggesting that the ASA agents could effectively promote osteogenic differentiation of ADSCs for a long period. With the increase in ASA concentration from 10 to 100 μg/ml (Zhang et al., 2022), mRNA expression levels of osteogenic markers were significantly increased compared with those of the control group, further demonstrating its osteoinductive effects on osteogenic differentiation *in vitro*.

## Conclusion

In summary, we developed an aspirin-based GelMA-CS DN hydrogel with sustained drug release behavior in solutions, which could not only regulate the microenvironment for supporting cell viability but also promote cell growth and proliferation to facilitate bone regeneration. This GelMA-CS@ASA DN hydrogel possessed porous structures, good stability, and satisfactory biological and mechanical properties. The cytotoxicity assay indicated excellent cell proliferation capacity, while the sustained ASA release *in situ* could regulate the microenvironment to promote osteoblast differentiation. *In vitro* results further verified that this kind of hydrogel was capable of facilitating bone regeneration. We believe this finding may provide a promising option for developing translational ASA formulation and construction of multifunctional tissue engineering scaffolds with controlled drug delivery in future.

## Data Availability

The raw data supporting the conclusion of this article will be made available by the authors, without undue reservation.
